# A non-linear relationship between lesion length and risk of recurrent cerebral ischemia after stenting for symptomatic intracranial stenosis with hemodynamic impairment

**DOI:** 10.3389/fneur.2023.1122708

**Published:** 2023-04-18

**Authors:** Xianjun Zhang, Wentao Gong, Zhen Meng, Guangwen Li, Peng Liu, Yong Zhang, Naidong Wang

**Affiliations:** ^1^Department of Neurology, The Affiliated Hospital of Qingdao University, Qingdao, Shandong, China; ^2^Department of Interventional Operating Room, The Affiliated Hospital of Qingdao University, Qingdao, Shandong, China

**Keywords:** intracranial stenosis, stent, lesion length, hemodynamic impairment, risk

## Abstract

**Background:**

Stent placement can be an effective treatment for patients with symptomatic intracranial stenosis (sICAS) and hemodynamic impairment (HI). However, the association between lesion length and the risk of recurrent cerebral ischemia (RCI) after stenting remains controversial. Exploring this association can help predict patients at higher risk for RCI and develop individualized follow-up schedules.

**Method:**

In this study, we provided a *post-hoc* analysis of a prospective, multicenter registry study on stenting for sICAS with HI in China. Demographics, vascular risk factors, clinical variables, lesions, and procedure-specific variables were recorded. RCI includes ischemic stroke and transient ischemic attack (TIA), from month 1 after stenting to the end of the follow-up period. Smoothing curve fitting and segmented Cox regression analysis were used to analyze the threshold effect between lesion length and RCI in the overall group and subgroups of the stent type.

**Results:**

The non-linear relationship between lesion length and RCI was observed in the overall population and subgroups; however, the non-linear relationship differed by subgroup of stent type. In the balloon-expandable stent (BES) subgroup, the risk of RCI increased 2.17-fold and 3.17-fold for each 1-mm increase in the lesion length when the lesion length was <7.70 mm and >9.00 mm, respectively. In the self-expanding stent (SES) subgroup, the risk of RCI increased 1.83-fold for each 1-mm increase in the lesion length when the length was <9.00 mm. Nevertheless, the risk of RCI did not increase with the length when the lesion length was >9.00mm.

**Conclusion:**

A non-linear relationship exists between lesion length and RCI after stenting for sICAS with HI. The lesion length increases the overall risk of RCI for BES and for SES when the length was <9.00 mm, while no significant relationship was found when the length was >9.00 mm for SES.

## 1. Introduction

Intracranial artery stenosis was found in approximately 46.6% of ischemic stroke patients in China and was associated with more severe strokes and longer hospital stays (1). The safety and efficacy of aggressive medical management over endovascular therapy were confirmed by the SAMMPRIS and VISSIT studies as the best treatment modality for symptomatic ICAS (2, 3). Currently, medical therapy, particularly for patients with hemodynamic impairment, still has a certain risk of recurrent cerebral ischemia (4, 5). In this group of patients, stent placement can further reduce this risk by improving cerebral hypoperfusion (6), and it is, therefore, considered a potential treatment modality.

For patients undergoing stent placement, there is a need to focus on perioperative safety and effectiveness during the long-term follow-up. The results of 14.7% of stroke or death within 30 days after stenting and 10% of stroke recurrence during 30 days to 3 years in the SAMMPRIS trial stent group were not satisfactory (2). Our previous study demonstrated that a well-designed operation strategy can lower the postoperative cumulative risk of stroke, TIA, and death after 30 days to 4.3% (7). However, during the long-term follow-up of our multicenter registry trial, approximately 9% of the incidence of target vessel-related recurrent ischemic stroke and TIA still requires thoughtful consideration.

Lesion length is one of the significant lesion characteristics that influence the safety and efficacy of stent placement in patients with symptomatic ICAS (8, 9). A *post-hoc* analysis of the SAMMPRIS trial revealed that lesion length is a potential risk predictor for 30-day stroke or death in sICAS patients with HI (10). However, the association between lesion length and the risk of recurrent cerebral ischemia 30 days after stenting remains unclear.

Our study, therefore, focused on the relationship between lesion length and the risk of recurrent cerebral ischemia in sICAS patients with HI, a population that may benefit from stent placement. We believe that the present study will aid in the identification of patients at high risk for recurrent ischemic episodes following stenting based on lesion length. It will also facilitate the individualization of follow-up management regimens that can be prepared in advance and that are aimed at improving the long-term clinical prognosis.

## 2. Material and methods

### 2.1. Study participants

The present study is a *post-hoc* analysis based on a prospective, multicenter registry study in China from September 2013 to January 2015. This registry study is designed to evaluate the short- and long-term efficacy and safety of stenting in patients with symptomatic ICAS and hemodynamics impairment in the real world. Details of the inclusion and exclusion criteria have previously been published. Written informed consent was available for all 300 patients who were enrolled. Among the aforementioned population, a total of 21 cases were excluded from this *post-hoc* analysis, including eight cases that were due to incomplete clinical baseline information and 13 cases with adverse endpoints (including stroke, TIA, and death) within 1 month after stenting. The final number of patients enrolled in the study was 279.

### 2.2. Definition of recurrent cerebral ischemia

This study focused on recurrent cerebral ischemia, including ischemic stroke and transient ischemic attacks in the blood supply area of the stented artery from 1 month after stenting to the end of the follow-up period. TIA is the sudden onset of neurological deficits due to cerebral ischemia, with completely reversible symptoms within 24 h. Ischemic stroke is defined as a new neurological disorder lasting more than 24 h and that could be confirmed by brain computed tomography (CT) or magnetic resonance imaging (MRI).

### 2.3. Stent selection and perioperative regimens

In this study, the types of the applied stent included a balloon-expandable stent (BES) (Apollo stent system, MicroPort Medical, Shanghai, China) and a self-expanding stent (SES) (Wingspan Stent System, Stryker, Maple Grove, Minnesota, USA). The surgeon individually selected the appropriate stent type according to the lesion characteristics, the recommended operation principles in the study protocol, and the clinical experience of the surgeon. The principles recommended the Apollo stent for Mori A type lesions and for patients with a well-passed arterial pathway, while the Wingspan stent was preferred for a tortuous arterial pathway, Mori C-type lesions, or lesions with a significant mismatch between proximal and distal diameters.

The patients received dual antiplatelet therapy (aspirin 100 mg/day and clopidogrel 75 mg/day) for a minimum of 5 days prior to surgery. If it is an emergency procedure and the patient is not taking clopidogrel, a single loading dose (300 mg of clopidogrel) will be administered. Moreover, the dual antiplatelet regimen was continued for 90 days following stent placement. At follow-up examinations, the management of vascular risk factors included: (1) blood pressure: target value of systolic blood pressure was <140 mmHg, and in the diabetic patients, it was <130 mmHg; (2) low-density lipoprotein: <70 mg/dL (1.81 mmol/L) or at least 50% less from the baseline; and (3) lifestyle factors: regular exercise, smoking cessation, and proper body mass index.

### 2.4. Study variables

Demographic variables, vascular risk factors, clinical variables, and lesion- or procedure-specific variables comprised the baseline variables. Before measuring lesion length based on digital subtraction angiography (DSA) images, the images were accurately calibrated using the following methods: (1) automatic calibration based on the digital subtraction angiography machine; (2) manual calibration based on the known diameter of the catheter located in the vessel; and (3) manual calibration based on a known diameter of the metal sphere located on the body surface. In addition, plaque surface morphology (PSM) was evaluated and confirmed by DSA, a subjective evaluation with no specified criteria. The evaluation method was based on a previous publication (11).

### 2.5. Statistical analysis

We evaluated the normality of continuous variables using the Kolmogorov–Smirnov test. Variables with a normal distribution were represented as mean ± standard deviation (SD), or otherwise by median (interquartile ranges, IQRs). Two independent samples *t*-test (normal distribution variables) or Mann–Whitney U-test (non-normal distribution variables) were used to assess the baseline difference between the BES and SES groups. Categorical variables were expressed as frequencies, and the percentages and differences between groups were tested by the chi-square test or Fisher's exact probability method. The conditions for the chi-square test and Fisher's exact test are as follows. The total number of samples (N) and the expected count (T) for each variable were computed. For dichotomous variables, Pearson's chi-square test was chosen when N ≥ 40 and all T ≥ 5; the continuously corrected chi-square test was chosen when N ≥ 40 and any of the minimum T's ranged from 1 to 5; and Fisher's exact test was chosen when (1) N ≥ 40 and two or more of the minimum T's ranged from 1 to 5 and when N < 40 or any of the minimum T < 1.

In the overall population and in subgroups that were divided by different types of stents, smoothing curve fitting was used to determine whether there was a non-linear relationship between lesion length and RCI. Segmented Cox regression analysis, logarithmic likelihood ratio (LRT) test, and Bootstrap resampling were performed to analyze the threshold effect between lesion length and RCI (12). We established three models in total: Model I was the one-line effect and Models II to III were the results of applying segmented COX regression analysis. Model II was not adjusted for variables, and Model III was adjusted for selected variables. The selection of the adjusted variables for Models I and III were based on the principle of a directed acyclic graph (DAG) (Supplementary Figure) (13) (excluding the intermediate variable, stent type, Mori type, lesion location, and plaque surface morphology) and on the aforementioned results, wherein the “change-in-estimate and backward elimination” method (14) was applied to continue selecting variables.

All statistical analyses were performed using the R statistical software version 3.4.3 (R Foundation) and the Empower Stats software (X&Y Solutions, Inc., Boston, USA). A two-sided *p* < 0.05 was considered to be statistically significant.

## 3. Results

### 3.1. Participant characteristics

A total of 279 cases were included in this study, with a median follow-up period of 24.73 months (IQR, 19.93–28.55). A total of 26 recurrent ischemic strokes or TIA events occurred during the follow-up period. The baseline characteristics of the overall population and the different subgroups by stent type are shown in Tables 1, 2. Among the total group, lesion length (mm) had a maximum of 17.00, a minimum of 1.80, and a median of 7.11 (IQR, 5.04–9.30). In the BES subgroup, the maximum was 15.02, and the minimum was 1.80. In the SES subgroup, the maximum was 17.00, and the minimum was 1.90. The median lesion length was significantly higher in the SES subgroup than in the BES subgroup (8.00 IQR 6.30–10.00 vs. 6.00, IQR 5.00–8.00, *p* < 0.001).

**Table 1:** Baseline categorical variables of patients in the overall group or stent subgroups.

	**Overall group**	**BES group**	**SES group**	
**Variables**	***N** =* **279**	***N** =* **139**	***N** =* **140**	***P*** **value**
	**No. (%)**	**No. (%)**	**No. (%)**	
Sex				0.554
Female	68 (24.37)	36 (25.90)	32 (22.86)	
Male	211 (75.63)	103 (74.10)	108 (77.14)	
History of Smoking				0.947
Never	125 (44.80)	62 (44.60)	63 (45.00)	
Current or former	154 (55.20)	77 (55.40)	77 (55.00)	
History of Drinking				0.417
No	170 (60.93)	88 (63.31)	82 (58.57)	
Yes	109 (39.07)	51 (36.69)	58 (41.43)	
History of Hypertension				0.818
No	78 (27.96)	38 (27.34)	40 (28.57)	
Yes	201 (72.04)	101 (72.66)	100 (71.43)	
History of Diabetes mellitus				0.408
No	197 (70.61)	95 (68.35)	102 (72.86)	
Yes	82 (39.39)	44 (31.65)	38 (27.14)	
History of Hyperlipidemia				0.754
No	162 (58.06)	82 (58.99)	80 (57.14)	
Yes	117 (41.94)	57 (41.01)	60 (42.86)	
History of Stroke or TIA				
No	146 (52.33)	72 (52.17)	74 (52.86)	0.860
Yes	133 (47.67)	67 (48.20)	66 (47.14)	
Qualifying Event				0.592
TIA	126 (45.16)	65 (46.76)	61 (43.57)	
Stroke	153 (54.84)	74 (53.24)	79 (56.43)	
mRS Score				0.195
0	92 (32.97)	50 (35.97)	42 (30.00)	
1	144 (51.61)	73 (52.52)	71 (50.71)	
2	41 (14.70)	16 (11.51)	25 (17.86)	
3	2 (0.72)	0 (0.00)	2 (1.43)	
Lesion angulation				0.103
< 45 degree	253 (90.68)	130 (93.53)	123 (87.86)	
>45 degree	26 (9.32)	9 (6.47)	17 (12.14)	
Plaque surface morphology				0.083
Smooth	130 (46.59)	72 (51.80)	58 (41.43)	
Irregular or ulcerated	149 (53.41)	67 (48.20%)	82 (58.57)	
Eccentric stenosis				0.433
No	110 (39.43)	58 (41.73)	52 (37.14)	
Yes	169 (60.57)	81 (58.27)	88 (62.86)	
Lesion location				0.078
Trunk	257(92.11)	132 (94.96)	125 (89.29)	
Opening or Bifurcation	22(7.89)	7 (5.04)	15 (10.71)	
Mori Type				0.004
Mori A or B	226 (81.00)	122 (87.77)	104 (74.29)	
Mori C	53 (19.00)	17 (12.23)	36 (25.71)	
Symptomatic artery				< 0.001
ICA	38 (13.62)	22 (15.83)	16 (11.43)	
MCA	86 (38.82)	28 (20.14)	58 (41.43)	
BA	87 (31.18)	45 (32.37)	42 (30.00)	
VA	68 (24.37)	44 (31.65)	24 (17.14)	

TIA, transient ischemic attack; ICA, intracranial carotid artery; MCA, middle cerebral artery; BA, basilar artery; VA, vertebral artery.

**Table 2:** Baseline continuous variables of patients in the overall group or stent subgroups.

	**Median (IQR) or Mean** ±**SD**			
**Variables**	**Overall group**	**BES Group**	**SES Group**	***P*** **value**
	***N** =* **279**	***N** =* **139**	***N** =* **140**	
Age, y	58.43 ± 9.75	59.95 ± 8.67	56.92 ± 10.52	0.009
BMI, Kg/m^2^	25.58 ± 3.05	25.77 ± 3.16	25.39 ± 2.94	0.310
NIHSS Score	0 (0–2)	0 (0–2)	0 (0–2)	0.245
Time of QE to stent, d	21.00 (10.00–34.00)	20.38 (9.00–35.27)	22.00 (11.80-−32.25)	0.779
Lesion length, mm	7.11 (5.04–9.30)	6.00 (5.00–8.00)	8.00 (6.30–10.00)	< 0.001
Stenosis, %	83.98 ± 9.05	83.59 ± 10.15	84.37 ± 7.83	0.475
Residual stenosis, %	10.00(0–10.00)	5.00 (0.00–10.00)	10.00 (5.00–20.00)	< 0.001

BMI, body mass index; QE, qualifying event.

### 3.2. Association of lesion length with the RCI risk

**Figure 1:**
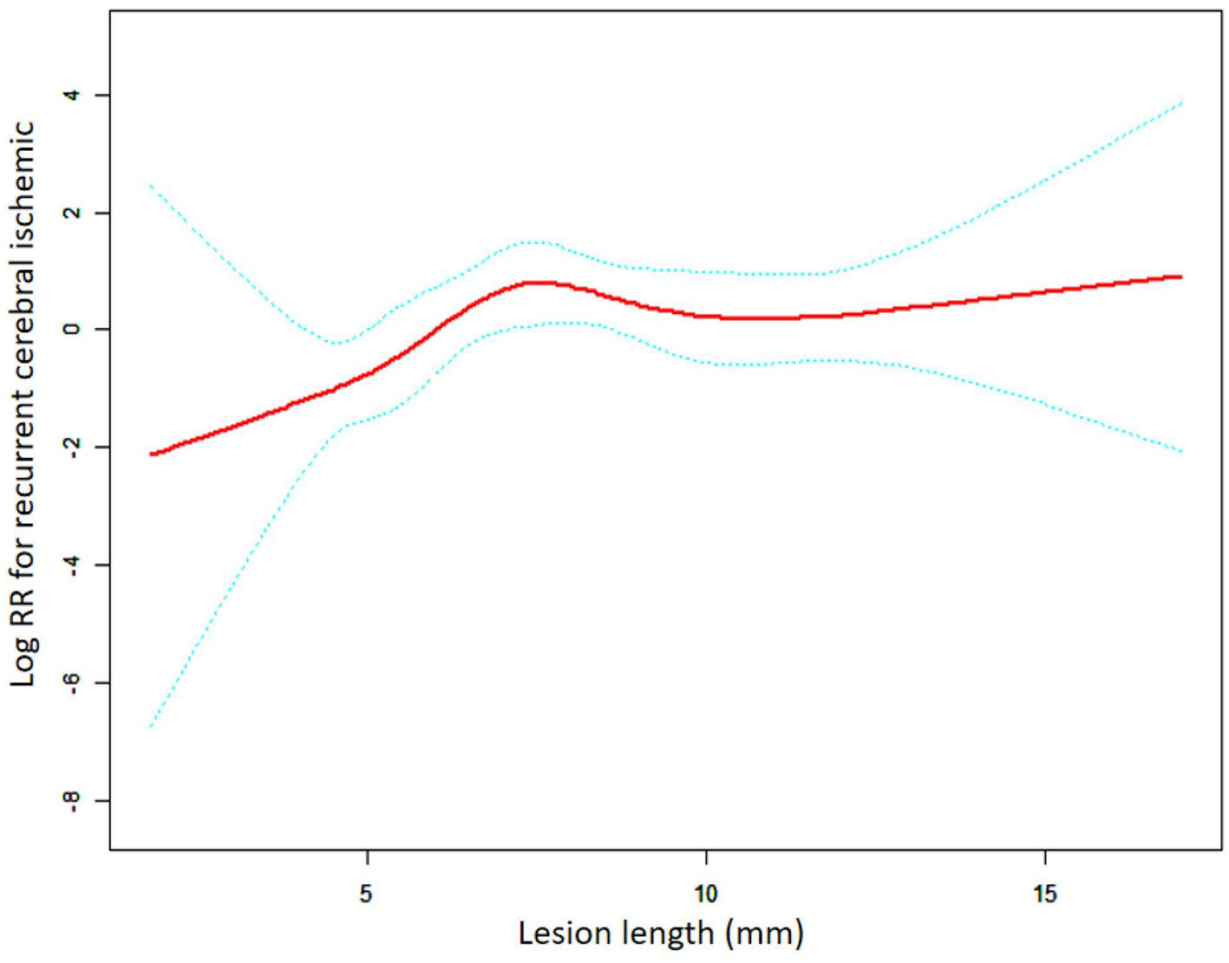
A non-linear association between lesion length and recurrent cerebral ischemic in the overall group. The red solid line indicates the smooth curve fit between lesion length and RCI. The blue dotted line represents the 95% confidence interval of the fit (RR, relative risk).

Among the overall group, there was a non-linear relationship between lesion length and RCI risk (Figure 1). The threshold effect analysis for the aforementioned nonlinear relationship is presented in Table 3. The turning point was verified to be 7.00 mm in length. In patients with a lesion length <7.00 mm, the risk of RCI increased 1.88-fold for each 1.00-mm increase in the lesion length (adjusted HR = 1.88, 95% CI: 1.10–3.22, *p* = 0.022). Furthermore, when the lesion length was >7.00 mm, the continued increase in length was not associated with an increase or decrease in RCI risk (adjusted HR = 0.94, 95% CI: 0.75–1.19, p = 0.613). For the LRT test, *p* = 0.031, which showed that a non-linear relationship exists between the lesion length and the RCI risk. Subgroup analysis was based on the stent type.

**Table 3:** Threshold effect analysis of the relationship between lesion length and RCI in the overall group.

	**Length**	**HR (95%CI)**	***P* value**
**Model I**			
One line effect		1.13 (0.98, 1.29)	0.090
**Model II**			
Turning point (mm)	7.00		
< 7.00		1.50 (0.97, 2.31)	0.069
>7.00		0.65 (0.38, 1.13)	0.125
LRT test			0.025^*^
**Model III**			
Turning point (mm)	7.00		
< 7.00		1.88 (1.10, 3.22)	0.022^#^
>7.00		0.94 (0.75, 1.19)	0.613
LRT test			0.031^*^

Model I: Linear analysis and adjusted for age, mRS score, symptomatic artery, and residual stenosis.

Model II: Non-linear analysis and no variables adjusted.

Model III: Non-linear analysis and adjusted for the same variables as Model I.

^*^LRT (likelihood ratio) test (p-value<0.05) indicates Models II and III are significantly different from Model I and presents a non-linear relationship.

^#^Indicates statistically significant HR values in the threshold effects analysis for subgroups.

**Figure 2:**
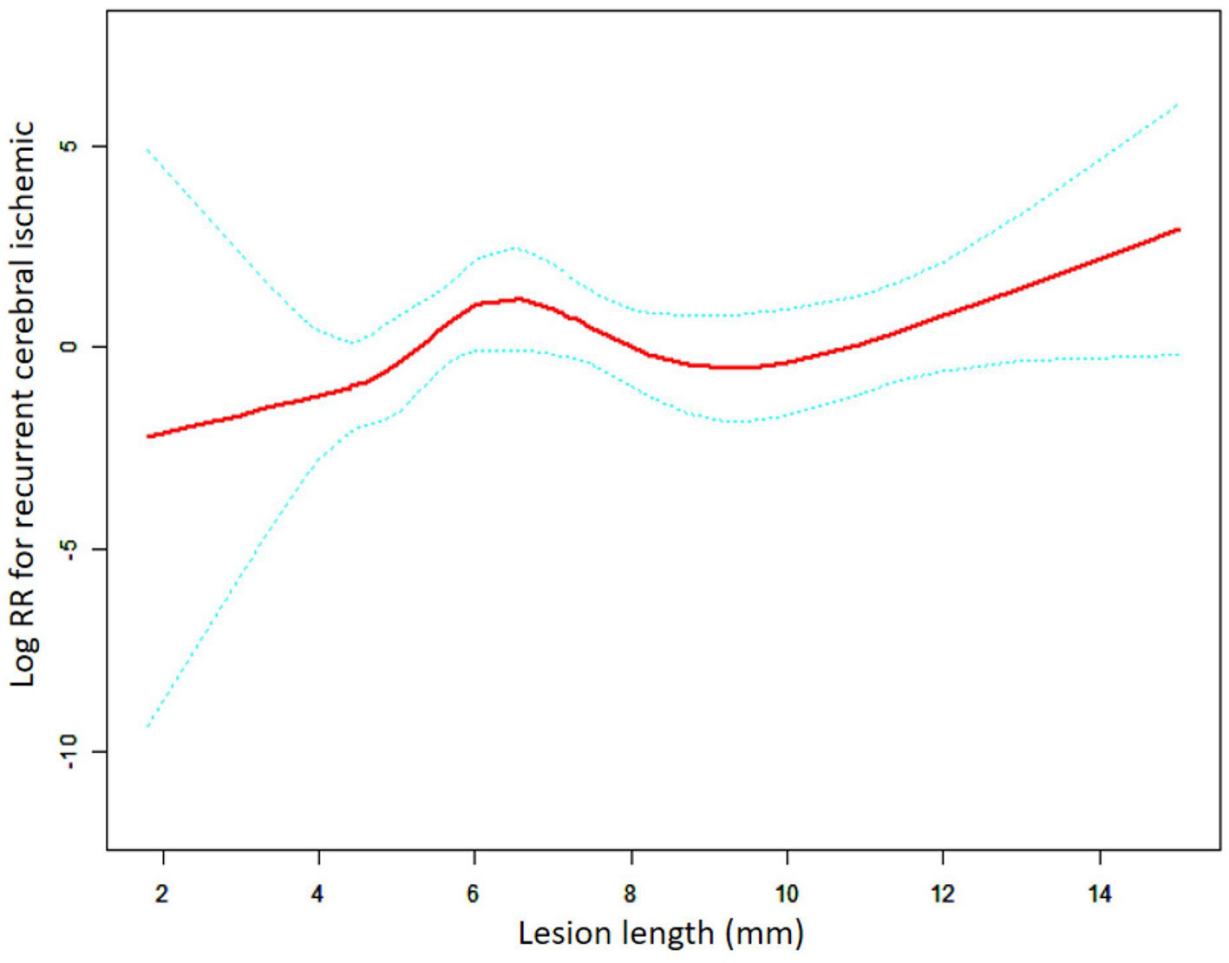
A non-linear association between lesion length and recurrent cerebral ischemic in the BES group. The red solid line indicates the smooth curve fit between lesion length and RCI. The blue dotted line represents the 95% confidence interval of the fit (RR, relative risk).

**Figure 3:**
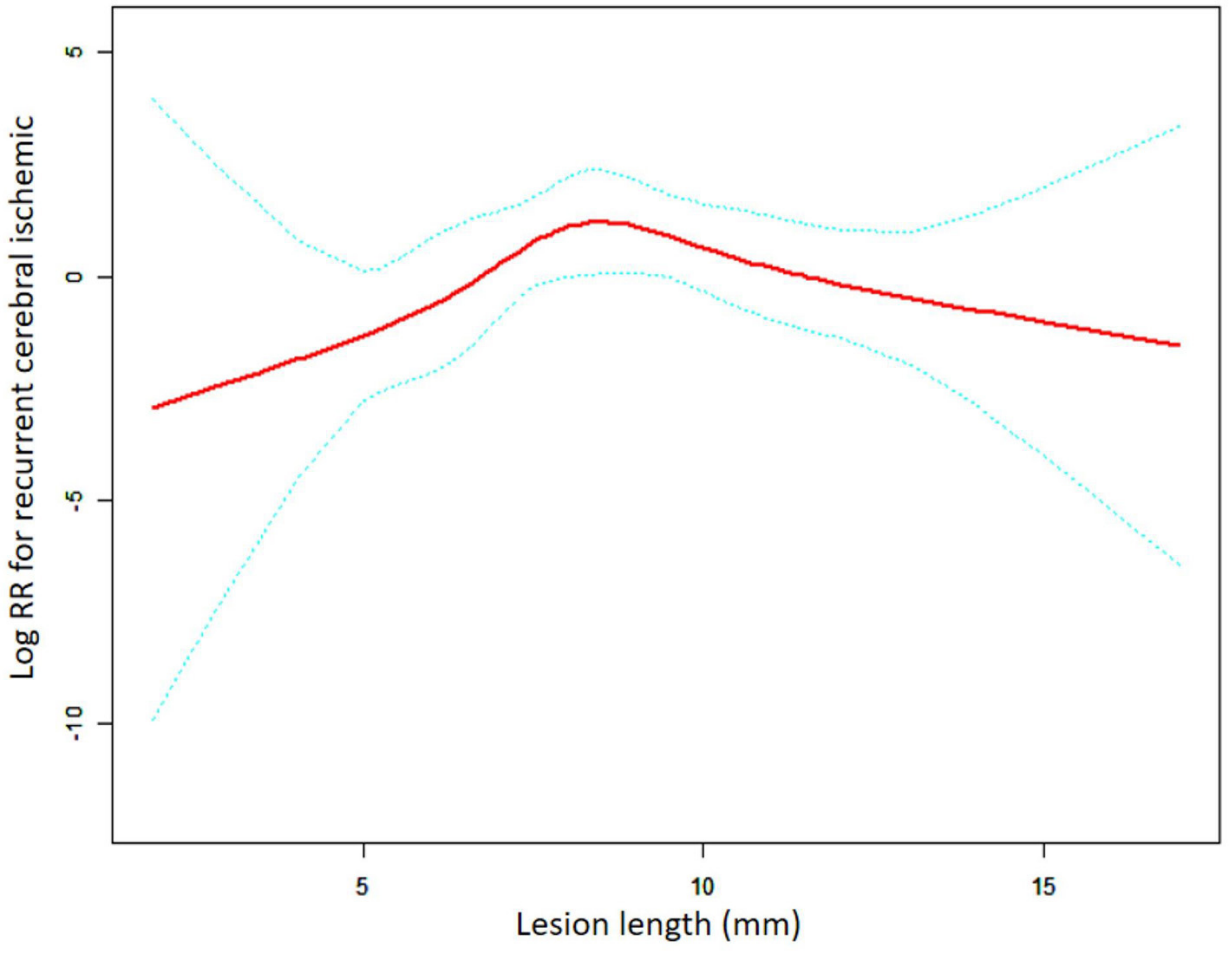
A non-linear association between lesion length and recurrent cerebral ischemic in the SES group. The red solid line indicates the smooth curve fit between lesion length and RCI. The blue dotted line represents the 95% confidence interval of the fit (RR, relative risk).

This subgroup analysis evaluated the differences in the relationship between the lesion length and risk following the implantation of different stent types. As shown in Figures 2, 3, the aforementioned non-linear relationships vary depending on the subgroups of stents. Using the analytical method of the threshold effect, we identified two turning points in the balloon-expandable stent subgroup, 7.0 mm and 9.0 mm, respectively. After adjusting for the corresponding covariates and performing segmented Cox regression analysis, the results shown in Figure 2 and Table 4 were obtained. When the lesion length is <7.70 mm or >9.00 mm, the risk of RCI increases 2.17 (adjusted HR = 2.17, 95% CI: 1.06–4.46, p = 0.034) or 3.17 (adjusted HR = 3.17, 95% CI: 1.18–8.50, *p* = 0.022) times for each 1.00-mm increase in length, respectively. However, there was no difference in risk between 7.70 mm and 9.00 mm in length (adjusted HR = 0.10, 95% CI: 0.01–8.61, *p* = 0.314). The results of the LRT test (*p* = 0.037) indicated the existence of a non-linear relationship.

**Table 4:** Threshold effect analysis of the relationship between lesion length and RCI in the BES group.

	**Length**	**HR(95%CI)**	***P* value**
**Model I**			
One line effect		1.17 (0.92, 1.49)	0.194
**Model II**			
Turning point (mm)	7.70,9.00		
< 7.70		1.48 (0.82, 2.70)	0.196
7.70–9.00		0.28 (0.02, 5.83)	0.409
>9.00		2.03 (0.88, 4.67)	0.097
LRT test			0.282
**Model III**			
Turning point (mm)	7.70,9.00		
< 7.70		2.17 (1.06, 4.46)	0.034^#^
7.70–9.00		0.10 (0.01, 8.61)	0.314
>9.00		3.17 (1.18, 8.50)	0.022^#^
LRT test			0.037^*^

Model I: Linear analysis and adjusted for age, mRS score, symptomatic artery, residual stenosis, and history of hypertension.

Model II: Non-linear analysis and no variables adjusted.

Model III: Non-linear analysis and adjusted for the same variables as Model I.

^*^LRT (likelihood ratio) test (p-value < 0.05) indicates Model III is significantly different from Model I and presents a non-linear relationship.

^#^Indicates statistically significant HR values in the threshold effects analysis for subgroups.

In the self-expanding stent subgroup, the results were obtained using the same method of analysis as the one employed for the balloon-expandable stent subgroup (Figure 3, Table 5). The non-linear relationship that was assessed using the LRT test (*p* = 0.013) remained the same. The turning point was confirmed to be 9.00 mm in length, and the risk of RCI significantly increased with length in patients with lesions length <9.00 mm (adjusted HR = 1.83, 95% CI: 1.04–3.23, *p* = 0.035). For patients with lesion length >9.00 mm, there was a gradual tendency for the RCI risk to decrease with the increase in length; however, no statistical significance was found (adjusted HR = 0.61, 95% CI: 0.33–1.12, *p* = 0.111).

**Table 5:** Threshold effect analysis for the relationship between lesion length and RCI in the SES group.

	**Length**	**HR (95%CI)**	***P* value**
**Model I**			
One line effect		1.09 (0.87, 1.35)	0.469
**Model II**			
Turning Point (mm)	9		
< 9		1.50 (0.97, 2.31)	0.069
>9		0.65 (0.38, 1.13)	0.125
LRT test			0.025^*^
**Model III**			
Turning Point (mm)	9		
< 9		1.83 (1.04, 3.23)	0.035^#^
>9		0.61 (0.33, 1.12)	0.111
LRT test			0.013^*^

Model I: Linear analysis and adjusted for symptomatic artery, residual stenosis, and eccentric stenosis.

Model II: Non-linear analysis and no variables adjusted.

Model III: Non-linear analysis and adjusted for the same variables as Model I.

^*^LRT (likelihood ratio) test (p-value<0.05) indicates that Models II and III are significantly different from Model I and presents a non-linear relationship.

^#^Indicates statistically significant HR values in the threshold effects analysis for subgroups.

## 4. Discussion

Currently, few studies have focused on the relationship between the lesion length of intracranial arterial stenosis and the risk of recurrent cerebral ischemia after stent placement. Our study is the first to confirm a nonlinear correlation between lesion length and RCI utilizing curve fitting and threshold effects analysis to identify correlative differences in subgroups of patients with different types of applied stents.

Mori et al. (15) were the first to suggest that the lesion length or morphology may affect the safety of balloon angioplasty of intracranial artery stenosis and classified the lesions into three types: type A (length <5 mm, concentric or moderate eccentric lesions), type B (length in 5–10mm, extremely eccentric lesion), and type C (Length >10 mm, angular lesions, >90°, with proximal arterial tortuosity). Mori A (Type A) has the highest success rate (92%) for stenting and the lowest risk of postoperative death, stroke, or surgical bypass (8%), while Mori C (Type C) has the lowest success rate (33%) and the highest risk (87%). The safety of intracranial arteries' stenting in the perioperative period is also related to the Mori type of lesion. Jiang et al. (16) demonstrated that Mori A lesions have a lower risk of stroke and death within 30 days after stent placement compared to that of Mori B or C lesions. Considering the subjective evaluation of a lesion morphology (concentric or eccentric) by DSA images (17), in our study, the assessed lesion characteristic was associated with the lesion length, which can be automatically measured and calibrated in various ways, and therefore, the results obtained were more objective, accurate, and credible.

Most of the present studies on lesion length and the prognosis of endovascular treatment for sICAS are primarily concerned with perioperative safety; however, the results of these studies are still controversial. Kurre et al. (18) included a total of 372 sICAS stenting cases and divided the lesion length into three groups (<5, 5–10, and >10 mm) and found only a trend of higher incidence with the increase in length in non-disabling events during hospitalization, but it was not statistically significant. Another study reported no significant differences in perioperative success or complications between lesion lengths of <7 mm and >7 mm (19). Ali et al. (17) found a decreased rate of stroke within 30 days following stenting for short lesions (<5 mm) versus medium-length lesions (>5 mm); however, this difference was not statistically significant. Nevertheless, in a *post-hoc* analysis from the SAMMPRIS study (10), lesion length was predictive of perioperative higher risk (adjusted OR 1.20, 95% CI: 1.02 to 1.39) but with limited predictive power (area under curve 0.606) for patients with self-expanding stents.

We hypothesized that the following factors contributed to the discrepancy and inaccuracy of the aforementioned research findings: (1) A non-linear association may exist between the lesion length and the study outcome, and thus, the association may not be correctly analyzed when applying linear regression methods; (2) transforming the continuous variable of lesion length into a categorical variable may result in the loss of some of the potential characteristics of this variable; (3) none of the aforementioned articles with negative results considered the effect of different stent types on the non-linear relationship, and a combination of self-expanding and balloon-expanding stents were included and analyzed; and (4) the definition of an endpoint event, which includes one or more strokes, TIAs, or deaths, differs among clinical studies.

In our study, the correlation between lesion length and clinical outcomes was precisely characterized and analyzed by applying curve fitting and threshold effects analysis to the continuous variable of the lesion length, as well as by performing subgroup analysis of different stent types. In addition, we chose RCI rather than perioperative complications as the clinical outcomes for the following reasons: (1) According to the protocol of our registry study, the endovascular treatment strategy individualized the stent type selection by different Mori types (lesion length included), which has been shown to effectively reduce perioperative complications to 4.3%; (2) given the high risk of RCI in our registry trial (nearly 9%), it was essential to study the association between lesion length and RCI to further identify high-risk groups and refine the follow-up plan and ultimately improve the long-term prognosis of patients.

For the first time, this study demonstrates a non-linear association between lesion length and RCI following stent placement during a long-term follow-up period in patients with sICAS and HI and the similarities and differences of this association among subgroups of stent type.

In the BES group, there was an overall trend of increased RCI risk with increasing lesion length (length < 7.70 mm: HR = 2.17, 95% CI: 1.06–4.46. length > 9.00 mm: HR = 3.17, 95% CI: 1.18–8.50), whereas a similar finding was observed in patients with a lesion length of <9.00 mm in the SES group (HR = 1.83, 95% CI = 1.04–3.23). In-stent restenosis (ISR) is a complication of stenting that can lead to RCI and is a crucial factor influencing the long-term prognosis following stenting (20). A *post-hoc* analysis of the SAMMPRIS indicated that after stent placement (21), ISR occurs in 66.7% or 80% of patients who had an ischemic stroke or TIA, respectively, during the follow-up. The lesion length is closely associated with in-stent restenosis of the intracranial artery (22, 23). First, during balloon angioplasty and stenting, the degree of endothelial damage is aggravated by the increasing lesion length, which subsequently induces a more intense inflammatory response and thrombosis, thus promoting the initiation and progression of ISR (24, 25). Second, the parent artery tortuosity, which is more prevalent in longer lesions, is not favorable to precise stent positioning and affects the degree of fit between the stent and the vascular wall, thereby leading to incomplete stent apposition and expansion and ultimately ISR (26–28). Furthermore, longer lesions frequently indicate greater plaque burden which may increase the likelihood of smooth muscle cell proliferation and migration to the neoplastic endothelium, resulting in hyperproliferation and negative remodeling of the endothelium and, hence, early ISR (<1 year) (23, 25). For late ISR (>1 year), the main pathophysiological mechanism is neoatherosclerosis, which is more common in patients with long lesions and atherosclerotic risk factors (29).

Our study further revealed that when the lesion length was >9.00 mm, the relationship between the lesion length and RCI was completely different in the BES and SES groups. In the BES group, the lesion length remained a risk factor for RCI (HR = 3.17, 95% CI: 1.18–8.50, *p* = 0.028), whereas there was no significant correlation between lesion length and RCI (HR = 0.54, 95% CI: 0.27–1.07, *p* = 0.079) in the SES group. We assume that the good flexibility of the SES still allows for adequate stent adherence to the lumen when used for longer or more tortuous lesions compared to shorter lesions. Submaximal angioplasty is typically performed prior to SES release to minimize intimal damage (30). Therefore, there is no significant increase in the incidence of ISR throughout the follow-up period in long lesions treated with SES compared to short lesions. Another study suggested that ISR associated with SES (Wingspan stent) more often begins in localized short lesions, and therefore, for lesions with the same length of the applied stent, ISR is likely to occur in those with a relatively shorter length and within a certain range (31). The event explains why the RCI risk does not rise with a further increase in the lesion length in the SES group. Moreover, the overall stress (axial plaque stress, APS) exerted by blood flow on the surface of neoplastic plaques in the ISR region is one of the key elements that trigger plaque rupture (32). A negative correlation between APS and lesion length has been identified, suggesting that the incidence of plaque rupture may be greater in short and focal lesions than in diffuse lesions (33). There is another possible explanation for why a higher risk of RCI was not observed in the SES group when the lesion length is >9 mm and continued to increase. Nevertheless, BES does not have as good flexibility compared to SES, which could lead to more vascular damage than SES with a similar diameter of dilation and more incomplete stent apposition, especially in long lesions. Our results further confirm that BES is unsuitable for long lesions and that an increase in the unit length results in additional RCI when the lesion length is >9.00 mm (HR = 3.17) compared to <7.70 mm (HR = 2.17).

Residual stenosis after stent placement is a significant predictor of recurrent stroke during the follow-up period. The presence of a calcified plaque in the lesion, inadequate balloon pre-dilation, and the application of self-expanding stents may lead to high residual stenosis in the lesion following stenting. Several studies have shown that higher residual stenosis after stenting is a risk factor for in-stent restenosis throughout the follow-up period, particularly when the residual stenosis is >30% (34, 35). As discussed previously, in-stent restenosis is closely associated with recurrent stroke. Thus, we believe that the higher residual stenosis after stenting is related to an increased risk of recurrent stroke over the follow-up period, which is important since it raises the incidence of long-term in-stent restenosis. Meanwhile, we added the variable of postoperative residual stenosis in our study and accounted for it as a confounding variable in the regression analysis to eliminate the effect of this variable on the outcome of the relationship between lesion length and RCI.

Notably, the variable and lesion angulation were omitted from the final regression analysis following the appropriate screening of variables in the overall population and subgroup analyses. This result appears to indicate that lesion angulation has no impact on RCI over the follow-up period; however, this may not be the case. The angle of the lesion reflects in part the artery's curvature at the lesion. Zhang et al. (36) observed that as arterial curvature increased, a local decrease in wall shear stress (WSS) and an increase in low-density lipoprotein (LDL) concentrations were seen in the inner artery wall of the curved segment. According to another study (37), the difference in arterial curvature generated by different stent shapes resulted in significant changes in the lesion's local WSS and LDL filtration rate. Low WSS and LDL penetrating the arterial wall from the circulation restrict nitric oxide (NO) release, hence triggering endothelial dysfunction (38, 39). The aforementioned process not only induced the formation of plaque on the stent surface (40) and the occurrence of restenosis but also delayed the appropriate endothelial coverage of the stent metal and raised the risk of late in-stent thrombosis. Throughout the follow-up period, either restenosis or in-stent thrombosis was a significant cause of RCI. Moreover, for preoperative lesions with large angles (curvature), substantially curved arterial morphology may persist following stent placement (especially with self-expanding stents). Thus, either the angle of the preoperative lesion or the morphology of the postoperative stent may have an effect on recurrent cerebral ischemia. During the clinical implementation phase, this study's data set did not gather postoperative stent morphological parameters, and the variable relating to lesion angle was presented as a dichotomous one rather than a continuous variable. Our analysis of the effect of lesion length on recurrent cerebral ischemia is limited by the absence of an adjustment for vascular curvature.

The Mori type is an important guide for the endovascular treatment of intracranial arterial stenosis, which has substantial implications for this study; therefore, the Mori type has been modified and supplemented for predicting long-term outcomes. It has been suggested that Mori C has a higher incidence of post-stent residual stenosis and in-stent restenosis than that of Mori A (9) and that it has a higher risk of long-term recurrent stroke than that of Mori A or B (41). Nevertheless, we consider that the Mori type has limitations when predicting long-term prognosis. First, risk differences may still exist for lesions with different characteristics in the same Mori type. For example, in the SES group, the lesions with angles <90° and lengths of 6 and 9 mm were classified as Mori B. The conventional approach was unable to compare the long-term prognosis of these two lesions, but our finding could further predict a better long-term prognosis for the 6 mm lesion. Second, different Mori-type lesions are likely to have similar long-term risks and prognoses. In the SES group, for instance, a Mori B lesion with a lesion length of 9 mm probably has a similar risk of RCI as a Mori C lesion with a length of 12 mm based on the findings of our study contrary to the findings of prior research studies. When semi-quantitatively assessing the risk of RCI during the follow-up period by Mori type, we should also focus on the evaluation in conjunction with the lesion length (as the continuous variable) as opposed to simply trichotomizing the lesion length (5, 5–10, and >10 mm) as the basis for Mori type. Consequently, our findings allow for a better quantitative and individualized prediction of long-term risk and management of patients.

Furthermore, the significance of our findings in guiding clinical practice includes the following: (1) Considering the HR values for different lesion length ranges in the BES subgroup, we should be particularly cautious when making clinical decisions on BES for lengthy lesions (>9 mm). Once BES is used for long lesions, we should develop a more rigorous follow-up plan for patients (including drug regimens and follow-up intervals) to reduce the risk of RCI. (2) In the SES subgroup, the risk of RCI in patients with long lesions (>9 mm) was no longer raised with increased length. Self-expanding stents may allow us to treat a range of long lesions without excessive concern about a worse prognosis due to the length of the lesion. The significance of this feature is not only to simplify the preoperative evaluation to a certain extent but also to potentially allow more patients with long lesions to have access to and benefit from endovascular treatment.

For the lesion length, some may argue that it is merely a surrogate of an array of other factors that potentially influence RCI, such as the severity of the disease, systemic or local inflammation, and flow reserve. Thus, further studies are needed to explore and analyze the pathophysiological mechanisms that influence lesion length, the pathological effects of different lesion lengths, and whether the effects could predict clinical outcomes (42).

In our study, there are other limitations that should be considered. First, the maximum lesion length was 17.00 mm, and the median length was 7.11 mm. Therefore, our findings, especially for SES, are still controversial for longer lesions (>17.00 mm), and thus, further studies are required. Second, the number of patients with a lesion length of >9.00 mm and the clinical endpoints were relatively small, which may have affected the statistical reliability of the results. Finally, the SES was the Wingspan stent system, which is the only SES approved by the FDA for the treatment of sICAS. With the increasing use of off-label SES for sICAS in the real world (43, 44) the relationship between the lesion length and RCI may vary between different types of SES.

## 5. Conclusion

Our study uncovered a non-linear correlation between the lesion length and RCI that is influenced by the stent type. In patients with self-expanding stents, there was a positive association between the lesion length and RCI when the lesion length was <9.00 mm. However, the RCI risk became larger with the increase in the lesion length in patients with balloon-expanding stents.

## Data availability statement

The original contributions presented in the study are included in the article/Supplementary material, further inquiries can be directed to the corresponding authors.

## Ethics statement

The studies involving human participants were reviewed and approved by the Ethics Committee of the Affiliated Hospital of Qingdao University. The patients/participants provided their written informed consent to participate in this study.

## Author contributions

XZ and WG: drafting of the manuscript, data organization and selection, data interpretation, and guarantor. ZM: data interpretation and manuscript proofreading. GL: drafting of the manuscript. PL: application of statistical software for data analysis. YZ: development and design of overall methodology and guarantor. NW: establishment of overall research goals and purposes, manuscript proofreading, and guarantor. All authors contributed to the article and approved the submitted version.
