# Supplementary material for: A non-linear relationship between lesion length and risk of recurrent cerebral ischemia after stenting for symptomatic intracranial stenosis with hemodynamic impairment
Source: Front Neurol. 2023 Apr 18;14:1122708. doi: 10.3389/fneur.2023.1122708 (PMC10151487; doi:10.3389/fneur.2023.1122708)
Supplement: Supplementary file 1 [file Data_Sheet_1.pdf]

Supplementary Material

1 Supplementary Figure

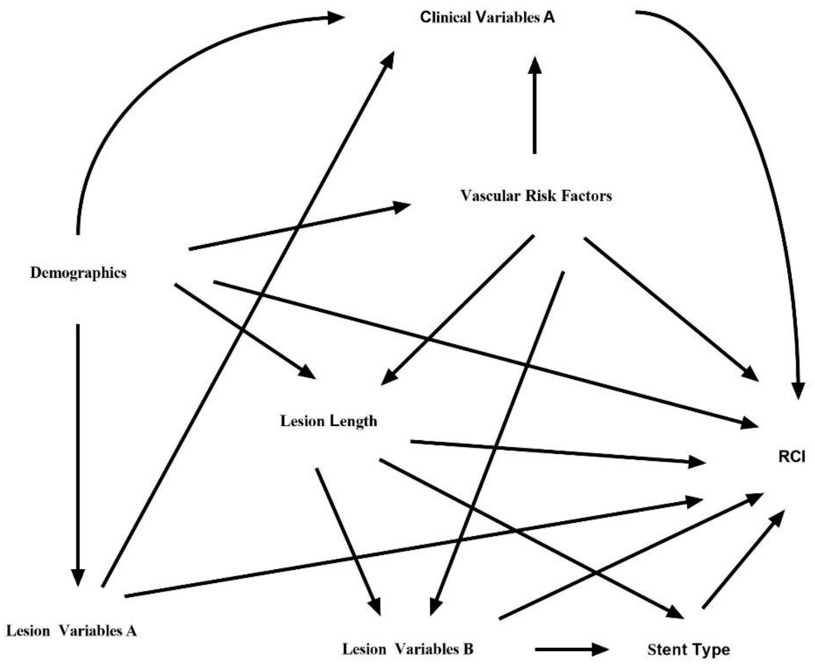

2 Supplementary Figure legend

Supplement Figure. Directed Acyclic Graph (DAG) illustrating variables selection.

Demographics：Sex, Age.

Vascular Risk Factors：BMI，History of smoking , Drinking, Hypertension, Diabetes mellitus, Hyperlipidemia.

Clinical Variables A：NIHSS Score，mRS Score，Qualifying Event，Time of QE to stent.

Lesion Variables A：Symptomatic artery，Lesion angulation，Stenosis，Residual stenosis, Eccentric Stenosis.

Lesion Variables B：Mori Type，Plaque surface morphology，Lesion location.

We did not adjust for intermediate factors (Lesion Variables B, stent type) in overall group and for intermediate factors (Lesion Variables B) in subgroups for the purpose of estimating the total effect of lesion length on recurrent cerebral ischemia (RCI).

Explanation for DAG

DAG (Directed Acyclic Graph) is a theory-driven independent variable screening method, which constructs a causal network based on the causal relationship of the theory, so as to find the appropriate independent variables to enter the model. By using DAG, we found that when the effect of lesion length on RCI was evaluated, lesion variable B and stent type were mediating variables, whereas other variables were confounding variables. Confounding variables need to be included in the multivariate regression analysis of correction variables to exclude confounding factors and mediating variables cannot be included. Because lesion length can indirectly affect RCI through mediating variables, this effect could not be included in a multifactorial regression analysis to exclude when assessing the overall effect of lesion length on RCI.
